# Arsenic-Induced, Mitochondria-Mediated Apoptosis Is Associated with Decreased Peroxisome Proliferator-Activated Receptor γ Coactivator α in Rat Brains

**DOI:** 10.3390/toxics11070576

**Published:** 2023-07-02

**Authors:** Bo Ding, Xinbo Ma, Yang Liu, Bangyao Ni, Siqi Lu, Yuting Chen, Xiaona Liu, Wei Zhang

**Affiliations:** Center for Endemic Disease Control, Chinese Center for Disease Control and Prevention, Harbin Medical University, Key Lab of Etiology and Epidemiology, Education Bureau of Heilongjiang Province & Ministry of Health (23618504), Harbin 150081, China; dingbo2020@foxmail.com (B.D.); 13259890828@163.com (X.M.); kkopj1988@163.com (Y.L.); 15082058335@163.com (B.N.); lusiqichn@163.com (S.L.); 18390356658@163.com (Y.C.)

**Keywords:** arsenic, PGC-1α, mitochondria, hippocampus, apoptosis

## Abstract

Chronic exposure to arsenic in drinking water damages cognitive function, and nerve cell apoptosis is one of the primary characteristics. The damage to mitochondrial structure and/or function is one of the main characteristics of apoptosis. Peroxisome proliferator-activated receptor γ coactivator α (PGC-1α) is involved in the regulation of mitochondrial biogenesis, energy metabolism, and apoptosis. In this study, we aimed to study the role of PGC-1α in sodium arsenite (NaAsO_2_)-induced mitochondrial apoptosis in rat hippocampal cells. We discovered that increased arsenic-induced apoptosis in rat hippocampus increased with NaAsO_2_ (0, 2, 10, and 50 mg/L, orally via drinking water for 12 weeks) exposure by TUNEL assay, and the structure of mitochondria was incomplete and swollen and had increased lysosomes, lipofuscins, and nuclear membrane shrinkage observed via transmission electron microscopy. Furthermore, NaAsO_2_ reduced the levels of Bcl-2 and PGC-1α and increased the levels of Bax and cytochrome C expression. Moreover, correlation analysis showed that brain arsenic content was negatively correlated with PGC-1α levels and brain ATP content; PGC-1α levels were negatively correlated with apoptosis rate; and brain ATP content was positively correlated with PGC-1α levels, but no significant correlation between ATP content and apoptosis has been observed in this study. Taken together, the results of this study indicate that NaAsO_2_-induced mitochondrial pathway apoptosis is related to the reduction of PGC-1α, accompanied by ATP depletion.

## 1. Introduction

Long-term consumption of water contaminated with arsenic is a growing public health issue, which may increase the risk of cancer, peripheral vascular disease, liver- and lung-related diseases, and diabetes, as well as neurological abnormalities, which affect more than 100 million people worldwide [1,2]. Nervous system diseases have always been the research focus, and the damage to the nervous system caused by arsenic in the environment has also been a widely shared concern. Arsenic accumulation reduces neurobehavioral functions, like intelligence and memory in children and adults. For example, epidemiological studies by Wasserman, G.A. proved that arsenic exposure impairs child’s memory [3]. Furthermore, research on Chinese adults and elderly people have indicated an association between excess arsenic intake from drinking water and cognitive impairment [4]. However, the mechanism of cognitive impairment induced by arsenic is still unclear.

The hippocampus is mainly responsible for learning, memory, cognition, mood, and other important functions related to behavior and intelligence. It is also one of the most vulnerable brain structures. The hippocampus is one of the main target organs of arsenic-induced cognitive impairment. It is reported that arsenic-induced rat hippocampal neurons are abnormally apoptotic and demonstrate damaged learning and memory abilities [5]. Arsenic induces neuron damage via multiple mechanisms, including mitochondria-mediated apoptosis. Our previous studies have also shown that arsenic causes apoptosis of rat cerebellar granule neurons, which is related to arsenic breaking the balance between Bax and Bcl-2 (B-cell lymphoma-2) [6,7,8]. The Bcl-2 family members promote apoptosis via hydrolysis, dephoshorylation, and other modifications. Apoptosis-promoting members change their intracellular localization from the cytoplasm to the mitochondrial membrane, where they open mitochondrial permeability transport pores, reduce mitochondrial membrane potential, and cause apoptotic signal molecules such as cytochrome C to enter the cytoplasm to activate the caspase family cascade reaction [9,10,11]. ATP is consumed in the process of apoptosis. For example, ATP is necessary for chromatin condensation in apoptotic cells [12]. The maintenance and reduction in intracellular ATP levels are systematically regulated during apoptosis [13]. ATP synthase on the inner mitochondrial membrane uses the energy of the mitochondrial membrane potential to synthesize ATP. When the mitochondrial permeability transport pores continue to open, the mitochondrial membrane potential decreases or even disintegrates completely, which reduces the production of ATP [14]. It is reported that arsenic exposure contributes to neuronal cell damage, accompanied by a decrease in ATP [15,16,17,18]. The change in ATP is related to the synthesis of mitochondria.

One of the most important transcriptional coactivators affecting mitochondrial biogenesis is peroxisome proliferator-activated receptor γ coactivator α (PGC-1α). PGC-1α has a selective activity to specific stimuli in different tissues, especially playing a major role in mitochondrial biogenesis and functional regulation and participating in cell death and stress responses. As previous studies have shown, inhibition of PGC-1α protein induces the disorder in mitochondrial dynamics and apoptosis [19] and activates the PGC-1α-related signal pathway to play a role in anti-oxidation and anti-apoptosis [6]. As reported, in the process of apoptosis of HT22 induced by ischemia/reperfusion in vitro, the expressions of PGC-1α and Bcl-2 were decreased, and the expression of Bax was up-regulated. On the contrary, by increasing PGC-1α and Bcl-2, the expression of Bax was down-regulated to reduce apoptosis [20]. In addition, the activation of PGC-1α can improve the cognitive ability of aging rats [21]. Therefore, it is necessary to explore the association between PGC-1α and ATP in arsenic-induced hippocampal nerve cell damage.

Hence, this present study aimed to explore the change in PGC-1α in arsenic-induced rat hippocampus damage and analyze the relationship between arsenic, PGC-1α, ATP, and apoptosis in rat brains exposed (NaAsO_2_, orally via drinking water) to different doses of arsenic (0, 2, 10, and 50 mg/L).

## 2. Materials and Methods

### 2.1. Materials and Reagents

Anti-PGC-1α (ab191838), Anti-Bcl-2 (ab196495), Anti-NeuN (ab177487), and Anti-Bax (ab32503) were purchased from Abcam Ltd. (Abcam, Cambridge, UK). Cytochrome C (#4272) was purchased from Cell Signaling Technology (Boston, MA, USA). The TUNEL Assay Kit was purchased with the In Situ Cell Death Detection Kit, POD (11684817910, Roche Applied Science). The ATP Content Assay Kit was purchased from Solarbio Life Science (BC0305, Beijing Solarbio Science & Technology Co., Ltd., Beijing, China). H&E staining solution (BL700B) was purchased from Biosharp Life Sciences. The immunohistochemistry kits (SV0002) were purchased from BOSTER (BOSTER Biological Technology Co., Ltd., Wuhan, China). The DAB color development solution (ZLI-9017) was purchased from Beijing ZhongShan JinQiao Company. The proteinase K solution (P1121) was purchased from Beijing Solarbio Technology Co., Ltd.

### 2.2. Animal and Experimental Designs

Male Wistar rats, 200–250 g, were purchased from Charles River Laboratory Animal Technology Co., Ltd. The rats were reared in a laboratory environment with a 12 h light/dark cycle, at 22 ± 2 °C and 50 ± 5% RH, with free access to drinking water and food. Food diets for the rats were purchased from Beijing Vital River Company. Forty rats were randomly divided into a control group, low dose group, medium dose group, and high dose group (*n* = 10). The rats in the four groups were exposed to 0, 2, 10, and 50 mg/L sodium arsenite (NaAsO_2_, CAS No. 7784-46-5, purchased from Sigma-Aldrich Chemical Company (Saint Louis, MO, USA), orally via drinking water) for 12 weeks. The protocol was approved by the ethics committee of Harbin Medical University (Ethical approval number: HRBMUECDC20210902).

### 2.3. Hematoxylin–Eosin (H&E) Staining

The H&E staining method [22] was used to detect brain pathological lesions. The brains were fixed with 4% paraformaldehyde and embedded in paraffin. The brain samples were cut into 5 μm thick sections. After that, the brain sections were dewaxed with xylene I and xylene II for 10 min each time. The dewaxed tissues were placed in 100% ethanol, 95% ethanol (I), 95% ethanol (II), 90% ethanol, 80% ethanol, 70% ethanol, and distilled water for 5 min each, then soaked in hematoxylin for 10 min and differentiated with 1% hydrochloric acid ethanol for 1–3 s. After that, they were stained with eosin for 7 min. Then, the slides were placed in 70% alcohol for 5 min, 80% alcohol for 5 min, 90% alcohol for 5 min, 95% alcohol for 5 min, 95% alcohol for 5 min, 100% alcohol (I) for 5 min, 100% alcohol (II) for 5 min, n-butanol (I) for 1 min, and n-butanol (II) for 1 min to dehydrate the sections. Then, the tissue slides were placed in xylene I and xylene II to make the sections transparent. Finally, the sections were sealed with neutral resin and observed under an Olympus Bx53 microscope.

### 2.4. Terminal Deoxynucleoitidyl Transferase-Mediated Nick End Labeling (TUNEL) Assay

According to the manufacturer’s instructions for the In Situ Cell Death Detection Kit, we detected the apoptosis of the CA1 region in hippocampus. The tissue sections were dewaxed and hydrated in xylene and different concentrations of ethanol. Then, the tissue was treated with 10 μg/mL proteinase K on ice for 5 min. Subsequently, the slides were incubated with 50 μL of the TUNEL reaction mixture at 37 °C for 60 min, followed by POD staining at 37 °C for 30 min. After DAB staining, the slides were stained with hematoxylin. Three slides were randomly selected from each group, and three fields (400×) were observed. TUNEL-positive cells were observed using an Olympus BX53 microscope. The positive cells in the TUNEL assay were brown or brown-yellow.

### 2.5. Observation of the Ultrastructure of Neurons in Hippocampus by Transmission Electron Microscopy (TEM)

The hippocampus was cut into about a 1 mm^3^ tissue block and fixed in 1.5 mL glutaraldehyde at 4 °C. The fixed tissue was also fixed with 1% osmic acid. Next, the tissues were placed in 30% acetone for 10 min, 50% acetone for 10 min, 70% acetone for 10 min, 90% acetone for 10 min, 100% acetone I for 10 min, 100% acetone II for 45 min, and 100% acetone III for 45 min. Then, it was placed in an acetone and pure resin solution for 1.5 h, and then embedded in pure resin and sections were prepared. Finally, the sections were stained with uranyl acetate and lead citrate, and observed and photographed by transmission electron microscopy.

### 2.6. Detection of Total Arsenic in Brain

Hydride generation atomic fluorescence spectrometry (HG-AFS) was used to detect the total arsenic content in rat brain tissues. The experimental method was performed in accordance with the Chinese national standard method (GB/T 5750.6-2006). Each sample was digested with a mixture of 900 μL concentrated nitric acid, 300 μL perchloric acid, and 300 μL concentrated sulfuric acid to digest 0.1 g brain tissue. Before detection, 1 mL of thiourea ascorbic acid and 1 mL of concentrated hydrochloric acid were added to the digested sample, which was then diluted to 10 mL with deionized water. The standard solution was prepared with a standard reference substance (GBW08611). A hydride generation atomic fluorescence spectrometer (AFS-933, Beijing Titan instruments, Beijing China) was used to detect the total arsenic level.

### 2.7. Immunohistochemistry

The immunohistochemical method [22] was used to detect PGC-1α, Bax, cytochrome C, and Bcl-2 expression. Brain paraffin sections were dewaxed and hydrated with xylene and ethanol, and then incubated with a 0.3% hydrogen peroxide solution for 15 min. The sections were placed in citric acid buffer (pH6.0), and the antigen was repaired using a microwave. The sections were blocked with 5% BSA at 37 °C for 30 min, then the sections were incubated with antibodies for PGC-1α (1:500), Bax (1:200), cytochrome C (1:150), and Bcl-2 (1:300) at 4 °C overnight. Next, the sections were incubated with goat anti-rabbit secondary antibody (BOSTER Biological Technology Co., Ltd., Wuhan, China) at 37 °C for 30 min. Then, the sections were stained using a DAB Staining Kit (BOSTER Biological Technology Co., Ltd., Wuhan, China), and then stained with hematoxylin. The positive cells were brown under a light microscope. From each group, three sections were randomly selected and three fields from each section were selected (400×). The average optical density of positive cells was observed, and the expression of PGC-1α, Bax, cytochrome C, and Bcl-2 was analyzed by IPP software.

### 2.8. Immunofluorescence Staining

The brain sections were dewaxed and hydrated. Next, the sections were incubated with PGC-1α antibody (1:50) and NeuN (1:200) overnight at room temperature. After washing with PBS, the sections were incubated with Cy3-marked 570 and FITC-marked 520 secondary antibodies (1:500) for two hours at room temperature followed by DAPI labeling and mounting in anti-fade medium. Images were collected with a Zeiss LSM-710 confocal microscope.

### 2.9. Detection of ATP Contents in Brain

According to the instructions of the kit, we detected the ATP content in brain tissues. Briefly, to about 0.1 g tissue, 1 mL extraction solution was added, homogenized in an ice bath, and centrifuged at 8000× *g* 4 °C for 10 min. To the supernatant, 500 uL chloroform was added, the solution was fully mixed, centrifuged at 10,000× *g* 4 °C for 3 min, and the supernatant was then processed as a liquid. After fully mixing, the absorbance value A1 at 340 nm was immediately measured for 10 s, and then it was placed into a 37 °C incubator for 3 min, and then the absorbance value A2 was measure. ATP content = 0.625 × ΔA determination/ΔA standard/tissue quality.

### 2.10. Statistical Analysis

SPSS17.0 statistical software was used to analyze the experimental data. The mean optical density, arsenic contents in the brain, and ATP contents in the brain were expressed as mean ± SD. The Image-Pro Plus software was used to analyze mean optical density. One-way analysis of variance (ANOVA) was used to analyze data, followed by the least significant test (LSD test). The correlation between the mean optical density, ATP contents in the brain, and arsenic in the brain was based on Pearson correlation analysis. Differences were statistically significant at *p* < 0.0.

## 3. Results

### 3.1. Arsenic Accumulated in the Brain and Induced Neuronal Apoptosis

**Figure 1:**
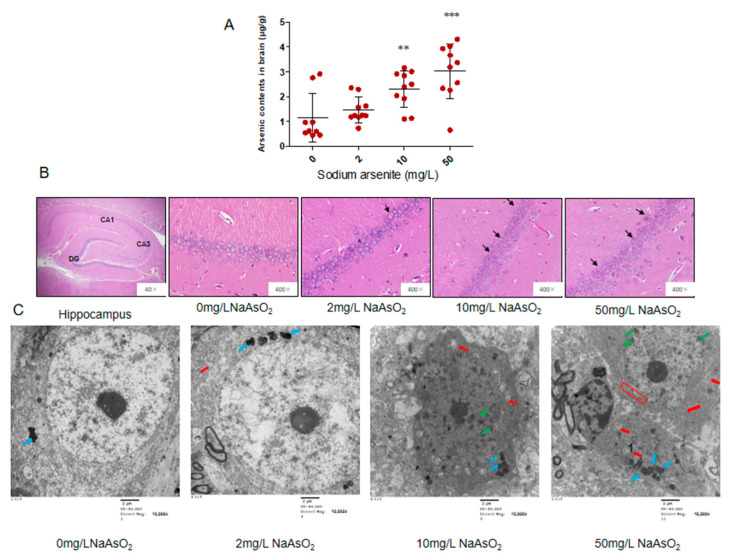
Arsenic contents, H&E staining, and ultrastructural changes in the hippocampal CA1 region in the rats exposed to arsenic. (**A**) Arsenic contents in brains of rats, *n* = 9–10. Red dots: brain arsenic levels in rats at different arsenic exposure levels. Results represent mean ± SD (μg/g). (**B**) H&E staining of rat hippocampal CA1 region, *n* = 9–10. Abnormal cells are indicated by black arrows. (**C**) The ultrastructure of neurons in the hippocampal CA1 region (scale bar = 2 μm), *n* = 3. Blue arrows: accumulated lipofuscin. Red arrows: vacuolated mitochondrion. Green arrows: chromatin condensation and margination. Red box: loss of integrity of the nuclear membrane. ** *p* < 0.01, *** *p* < 0.001 vs. control group.

We previously reported the body weights of the rats from NaAsO_2_ groups did not significantly change compared to control group [23]. In order to understand the accumulation of arsenic in brain, arsenic content was detected by atomic fluorescence spectrometry. The arsenic contents of the brain in the 0, 2, 10, and 50 mg/L NaAsO_2_ groups were 1.14 ± 0.98, 1.47 ± 0.51, 2.30 ± 0.75, and 3.03 ± 1.10 μg/g, respectively. These results showed that the arsenic level in the brain increased in the 2, 10, and 50 mg/L NaAsO_2_ groups, compared with the control group (*p* < 0.05). With the increase in NaAsO_2_ exposure dose, the brain arsenic content gradually increased. These results indicated that arsenic can accumulate in the brain (Figure 1A).

We used H&E staining to observe the pathological changes in the rat hippocampus. Under 400 times magnification, we can clearly see the hippocampal formation composed of CA1, CA3, and dentate gyrus (DG). The CA3 area of the hippocampus connects with the inferior dentate gyrus, extends inward into the dentate gyrus, and extends outward into the CA1 area. When magnified 400 times, the cells in the CA1 region of the control group were well organized, orderly, and closely arranged; the cytoplasm was evenly stained red, the nucleus was large, round, and dark blue, and the nucleolus was clear. Compared with the control group, the cells in each arsenic group showed different degrees of pathological changes, that is, with the increase of arsenic exposure, the cells in the hippocampal CA1 area gradually decreased, became irregularly arranged, and the shape was fuzzy, and some cells had an unclear nuclear boundary and swelling. The results are shown in Figure 1B.

The hippocampal CA1 region nerve cell ultrastructure observed by transmission electron microscopy showed that the cells in the control group had a clear outline, normal morphology, clear and complete nuclear membrane, uniform chromatin, large number of mitochondria, long circular or oblate shape, orderly arrangement of internal ridge, and complete structures. In the 2 mg/L NaAsO_2_ group, increased lipofuscin levels and swollen mitochondria were observed in hippocampal nerve cells, which indicated that the nerve cells were under stress. In the 10 mg/L NaAsO_2_ group, a slight disruption in the edge set of chromatin in the nucleus, swollen mitochondria, and reduced organelles were observed in nerve cells. In the 50 mg/L NaAsO_2_ group, hippocampal nerve cells showed more severe alterations, such as massive loss of organelles, disruption in the nuclear chromatin border set, loss of abnormal structural integrity of the nuclear membrane, and the nerve cells showed apoptotic features (Figure 1C).

### 3.2. TUNEL Analysis of the Hippocampal CA1 of Rats Exposed to Different Arsenic Concentrations

**Figure 2:**
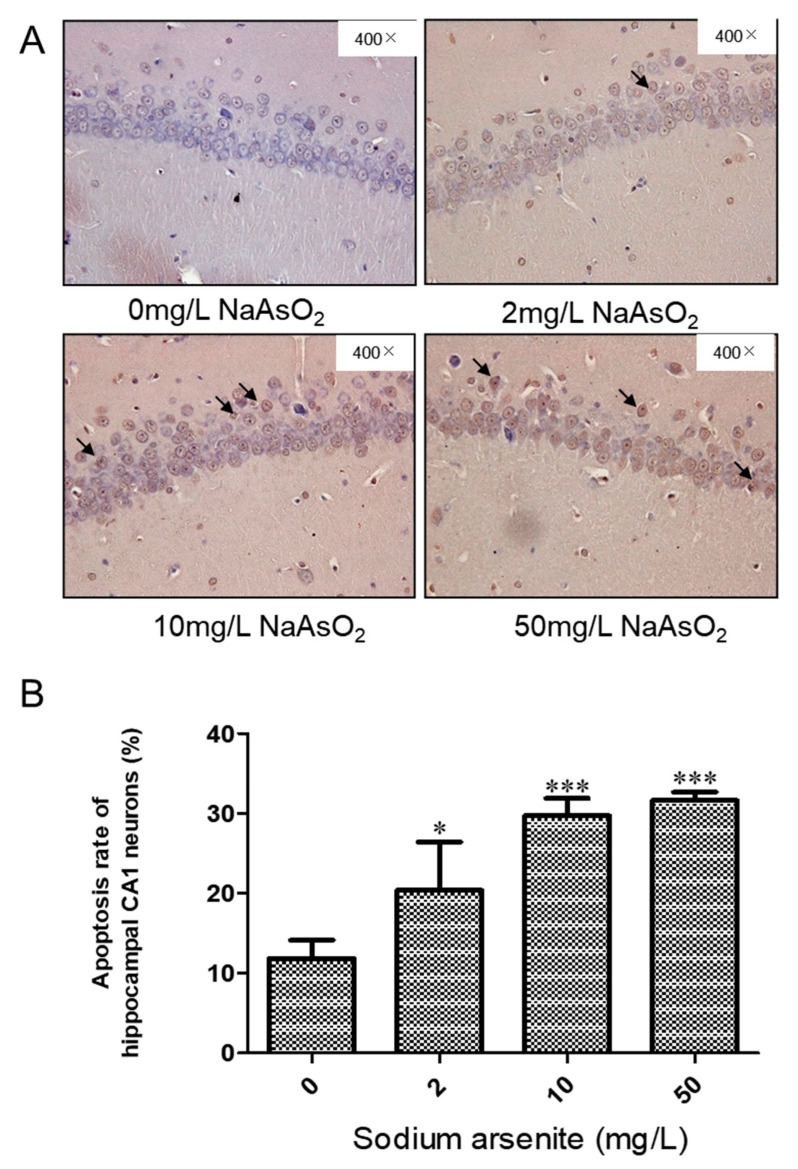
(**A**) Representative images of TUNEL-positive cells in the hippocampal CA1 region (magnification 400×). (**B**) Apoptotic ratio in the hippocampal CA1 region (results represent mean ± SD). * *p* < 0.05, *** *p* < 0.001 vs. control group, *n* = 3.

TUNEL staining was used to detect apoptosis in the hippocampi of rats exposed to different doses of arsenic. Cells with brown staining are TUNEL positive. The apoptosis rate was expressed as the percentage of TUNEL-positive cells in the total number of cells. The results showed that the apoptotic cell ratios of the hippocampus in the control group, 2, 10, and 50 mg/L NaAsO_2_ groups were 11.84 ± 2.27, 20.40 ± 6.01, 29.71 ± 2.18, and 31.63 ± 1.06, respectively. Compared with the control group, the apoptosis rate of the 2, 10, and 50 mg/L NaAsO_2_ groups was significantly increased (*p* < 0.05), but there was no significant difference in the apoptosis rates between the 10 mg/L and 50 mg/L NaAsO_2_ groups (Figure 2A,B). The above results indicated that NaAsO_2_ can induce apoptosis in nerve cells; in addition, the apoptotic cells induced by high doses of arsenic showed a plateau in this study.

### 3.3. Bcl-2, Bax, and Cytochrome C Expression in Hippocampal CA1 Region of Rats after Arsenic Exposure

**Figure 3:**
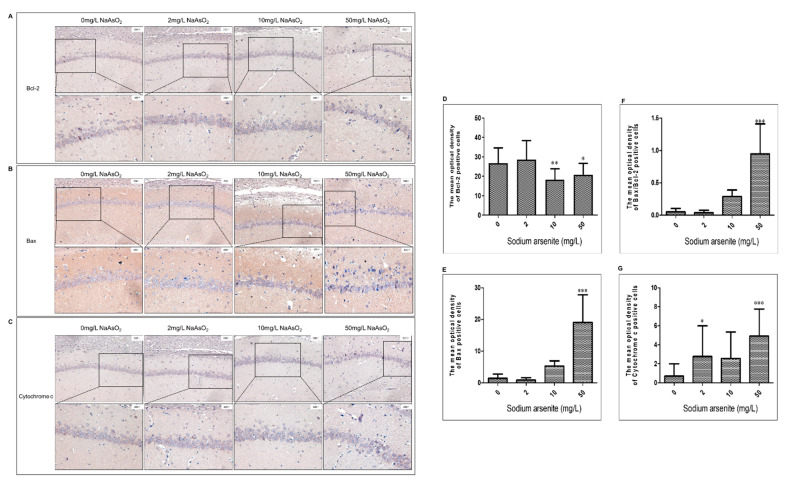
(**A**–**C**) Representative images of Bcl-2, Bax, and cytochrome C in the hippocampal CA1 region (magnification 200×, 400×), respectively. (**D**–**G**) Representative mean optical density analyses of Bcl-2, Bax, the ratio of Bax/Bcl-2, and cytochrome C (results represent mean ± SD). * *p* < 0.05, ** *p* < 0.01, *** *p* < 0.001 vs. control group.

Immunohistochemistry was used to analyze the effect of arsenic on Bcl-2, Bax, and cytochrome C expression. The immunohistochemical results showed that the expression of Bcl-2, Bax, and cytochrome C was positive in the hippocampal CA1 area of rat brains, which showed brown or brownish yellow granules in the cells. Compared with the control group, the level of Bcl-2 protein in the 10 and 50 mg/L NaAsO_2_ groups was significantly decreased (control group vs. 10 mg/L NaAsO_2_ group, *p* < 0.01; control group vs. 50 mg/L NaAsO_2_ group, *p* < 0.05; Figure 3A,D). Compared with the control group, the level of Bax protein in the 50 mg/L NaAsO_2_ group was significantly increased in a dose-dependent manner (*p* < 0.001; Figure 3B,E), whereas the Bax/Bcl-2 ratio increased significantly in the 50 mg/L NaAsO_2_ group (*p* < 0.001; Figure 3F). Furthermore, as shown in Figure 3G, the expression level of cytochrome C in the 2 and 50 mg/L NaAsO_2_ groups was significantly higher than that in the control group (*p* < 0.05). These results indicated that NaAsO_2_ induced mitochondrial pathway apoptosis.

### 3.4. PGC-1α Expression in Hippocampal CA1 Region of Rats Exposed to Different Arsenic Concentrations

**Figure 4:**
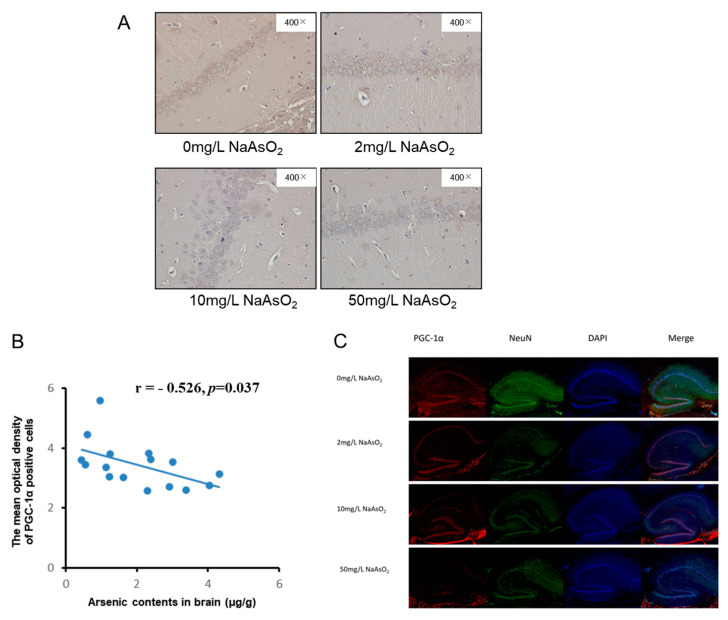
(**A**) and (**C**) Representative immunohistochemical (magnification 400×) and immunofluorescence (magnification 20×) images of PGC-1α in the hippocampal CA1 region, respectively. PGC−1α labeled in red, NeuN labeled in green, DAPI labeled in blue. (**B**) Representative image of correlation analysis between PGC-1α expression (the mean optical density of immunohistochemical staining) in hippocampal CA1 region and brain arsenic levels, *n* = 4.

Immunohistochemistry and immunofluorescence were used to observe the effect of arsenic on PGC-1α, a major regulator of mitochondrial biogenesis and function in the hippocampal CA1 region of rats. Immunohistochemistry showed that PGC-1α expression in hippocampal CA1 neurons was decreased after arsenic exposure (Figure 4A,C). In order to further analyze the relationship between PGC-1α and arsenic in the rat brain, we analyzed the correlation between PGC-1α and brain arsenic levels. The results showed that the expression of PGC-1α was negatively correlated with arsenic in rat brains, and the correlation coefficient was r = −0.526 (*p* < 0.05, Figure 4B), i.e., with the increase in arsenic in the rat brain, the PGC-1α expression gradually decreased. The immunofluorescence results showed that PGC-1α-positive cells expressed red granules in the cytoplasm and nucleus, and the PGC-1α-positive cells decreased in the hippocampal CA1 area in the rats exposed to arsenic (Figure 4C). These results showed that the decrease in PGC-1α was related to arsenic.

### 3.5. Correlation Analysis between Brain ATP, Arsenic, and PGC-1α Expression

**Figure 5:**
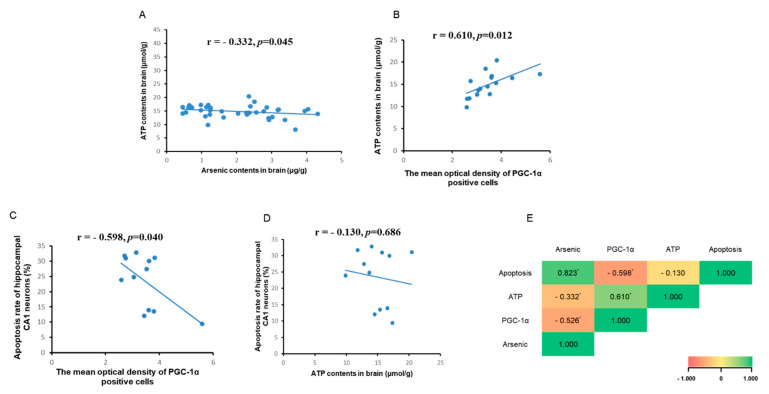
(**A**) Correlation analysis between ATP and arsenic contents in brain. (**B**) Correlation analysis between ATP content and PGC−1α immunohistochemical mean optical density. (**C**) Correlation analysis between apoptosis and PGC−1α immunohistochemical mean optical density. (**D**) Correlation analysis between apoptosis and ATP content. (**E**) Correlation analysis between arsenic, PGC−1α, ATP, and apoptosis in brain. * *p* < 0.05.

PGC-1α is a potent regulator of mitochondrial biogenesis and energy metabolism and affects the generation of ATP. Therefore, we detected the changes in brain ATP content in each group and analyzed the correlation between ATP, PGC-1α, and brain arsenic content and apoptosis. As shown in Figure 5A, the brain arsenic content showed a negative correlation with brain ATP, and the correlation coefficient r = −0.332 (*p* < 0.05), indicating that ATP content gradually decreased with the increase in brain arsenic content. As shown in Figure 5B,C, the correlation coefficient between ATP content and PGC-1α expression (immunohistochemical mean optical density) was r = 0.610, and the correlation coefficient between PGC-1α expression (average optical density of immunohistochemical) and apoptosis was r = −0.598, but there was no significant correlation between ATP content and apoptosis (Figure 5D).

## 4. Discussion

In this study, a rat model of arsenic exposure was established by orally administering the arsenic via drinking water. Our previous study reported that the arsenic levels in the serum and urine were both significantly increased in the arsenic exposure groups compared to the controls, and had no effect on body weight [23]. In this study, we detected brain arsenic contents in rats exposed to arsenic. These results showed that with the increase in arsenic exposure, the arsenic content in the brain increased, indicating that arsenic can be accumulated in the brain from drinking water. Then, the pathological changes of the neurons in the hippocampal CA1 area in each group were observed. In the control group, the neurons in the CA1 area were layered, arranged neatly and closely, with clear nucleoli and uniform cytoplasm. With the increase in arsenic exposure dose, disordered cell arrangement, blurred cell outlines, and some unclear nuclear boundaries and swollen cells were seen in the CA1 area. The ultrastructural results also showed that with the change in NaAsO_2_ concentration, the number of mitochondria in the hippocampal nerve cells decreased by varying degrees and the structure was incomplete. Meanwhile, with the increase in NaAsO_2_ concentration, the apoptosis rate increased significantly, but there was no markedly increased apoptosis between the 10 mg/L NaAsO_2_ group and 50 mg/L NaAsO_2_ group, suggesting that apoptotic cells in the hippocampal CA1 region reached saturation in the 50 mg/L NaAsO_2_ group in this study. These findings indicated that the rat model of arsenic exposure was successfully constructed, and suggests that arsenic causes neuronal apoptosis in the hippocampus CA1 area, accompanied by mitochondrial and other ultrastructural damage, which is consistent with the results of other studies [5,24].

Apoptosis is induced by endogenous and exogenous apoptotic pathways, with the mitochondrial apoptosis pathway as the executor of the endogenous apoptosis process. It is reported that arsenic, through changing apoptosis-related proteins Bax and Bcl-2 expression, damages rat hippocampi [25]. The regulation of apoptosis by the mitochondrial pathway involves many mechanisms, including the opening of MPTP [26], the release of apoptotic and anti-apoptotic proteins, causing or inhibiting the caspase cascade reaction, and destroying mitochondrial membrane structure [27]. Previous studies have showed that the release of apoptosis-promoting proteins, such as apoptosis initiation factor cytochrome C, apoptosis promoter and apoptosis effector caspase family proteins, SMAC family proteins, and Bax, induces apoptosis [28]. Bcl-2 and IAP family proteins can inhibit apoptosis by inhibiting the release of apoptotic proteins [29]. Apoptosis-related cascades are interrelated and produce apoptotic effects. Among the many apoptotic and anti-apoptotic gene families, the Bcl-2 family plays an important role in regulating mitochondrial membrane permeability, the cascade reaction of caspase family proteins, and changes in membrane potential. Among them, Bax can increase mitochondrial membrane permeability, form macromolecular channels, and make some macromolecules enter the cytoplasm, leading to the swelling of mitochondria and initiating the mechanism of apoptosis; in addition, it also acts with the transcription factor p53 to affect the membrane permeability of mitochondria, resulting in the loss of membrane potential [7,30]. Bcl-2 inhibits the production of excess reactive oxygen species, increases the load of calcium, blocks the cascade of caspases, and inhibits apoptosis [31]. In this study, Bax, Bcl-2, and cytochrome C expression in the hippocampal CA1 region of rats in each group was detected. The results showed that arsenic increased Bax and cytochrome C expression and decreased Bcl-2 expression. These results, which are consistent with other studies, show that arsenic induces neuronal apoptosis in the rat hippocampal CA1 region at least through the mitochondrial pathway [32].

Mitochondria produce adenosine triphosphate through oxidative phosphorylation and the electron transport chain, which produces byproducts such as ROS that can accumulate and induce oxidative stress [33], and the increase in expression of cytochrome C leads to apoptosis [34,35]. On the other hand, mitochondria can also counteract the damage caused by oxidative stress and apoptosis [33]. A previous study has shown that arsenic-induced nerve cell damage is also associated with PGC1-α, which is closely related to mitochondrial biogenesis [36]. PGC-1α is a main regulator of mitochondrial biogenesis including mitochondrial quality regulation mechanisms such as division, fusion, and mitosis and also affects mitochondrial function such as energy metabolism, anti-oxidative stress, and anti-apoptosis [37,38,39,40,41,42]. Some studies have shown that oxidative damage, mitochondrial biogenesis imbalance, and a decrease in PGC-1α and ATP are important injury characteristics of neurodegenerative diseases [43,44]. When agonists are used to promote PGC-1α expression, ATP increases, the mitochondria-dependent cell apoptosis pathway proapoptotic protein Bax is downregulated, and anti-apoptotic protein Bcl-2 is upregulated, alleviating the neuronal damage [45]. It was also reported that overexpression of PGC-1α decreased apoptosis [42,44]. In this study, the PGC-1α level in the hippocampus was decreased in arsenic-exposed rats. In order to further analyze the association between PGC-1α and brain arsenic, we analyzed the correlation between PGC-1α and arsenic; the result showed that arsenic has a negative effect on PGC-1α expression in the brain in rats exposed to arsenic. Then, we analyzed the effect of arsenic on the mitochondrial apoptosis pathway and PGC-1α expression in this study; the result showed that there was a negative correlation between apoptosis and PGC-1α, which suggested that PGC-1α plays an important role in arsenic-induced hippocampal neuronal cell apoptosis. Previous studies have shown that brain ATP content is regulated by PGC-1α, and the decrease of PGC-1α reduces the content of ATP [46,47,48]. In this study, there was positive correlation between PGC-1α and ATP contents. In addition, there was a negative correlation between arsenic in rat brains and ATP content in brain tissue, that is, arsenic in the brain decreased ATP content, but the results did not show an obvious relationship between the reduction in ATP and apoptosis, which may be due to the involvement of other mechanisms in the process of arsenic affecting energy metabolism. Some limitations of this study should be noted. The detailed mechanism needs further exploration in the future.

## 5. Conclusions

The present study found that arsenic induced rat hippocampal neuronal cell mitochondrial pathway apoptosis, accompanied by incomplete mitochondrial structure, a decrease in PGC-1α expression, and reduction in ATP. In addition, we also found that brain arsenic has negative effects on PGC-1α and ATP: PGC-1α expression and ATP contents in the brain were negatively correlated with the brain arsenic content; PGC-1α expression was negatively correlated with apoptosis in the hippocampal CA1 region; and PGC-1α expression was positively correlated with ATP content in the brain. However, no obvious linear relationship between ATP and apoptosis was observed, which may mean that the reduction in ATP is not the direct result of arsenic-induced nerve cell apoptosis. This study contributes to and provides novel insight into broader mechanisms by which arsenic injures the hippocampus.

## Data Availability

Not applicable.

## References

[1] Hong Y.S., Song K.H., Chung J.Y. (2014). Health effects of chronic arsenic exposure. J. Prev. Med. Public Health.

[2] Raju N.J. (2022). Arsenic in the geo-environment: A review of sources, geochemical processes, toxicity and removal technologies. Environ. Res..

[3] Wasserman G.A., Liu X., Parvez F., Ahsan H., Factor-Litvak P., Geen A.V., Slavkovich V., Lolacono N.J., Cheng Z., Hussain I. (2004). Water Arsenic Exposure and Children’s Intellectual Function in Araihazar, Bangladesh. Environ. Health Perspect..

[4] Liu J., Gao Y., Liu H., Sun J., Liu Y., Wu J., Li D., Sun D. (2017). Assessment of relationship on excess arsenic intake from drinking water and cognitive impairment in adults and elders in arsenicosis areas. Int. J. Hyg. Environ. Health.

[5] Sun H., Yang Y., Shao H., Sun W., Gu M., Wang H., Jiang L., Qu L., Sun D., Gao Y. (2017). Sodium Arsenite-Induced Learning and Memory Impairment Is Associated with Endoplasmic Reticulum Stress-Mediated Apoptosis in Rat Hippocampus. Front. Mol. Neurosci..

[6] Wang P., Zhao M., Chen Z., Wu G., Fujino M., Zhang C., Zhou W., Zhao M., Hirano S.I., Li X.K. (2020). Hydrogen Gas Attenuates Hypoxic-Ischemic Brain Injury via Regulation of the MAPK/HO-1/PGC-1a Pathway in Neonatal Rats. Oxid. Med. Cell. Longev..

[7] Wang Y., Bai C., Guan H., Chen R., Wang X., Wang B., Jin H., Piao F. (2015). Subchronic exposure to arsenic induces apoptosis in the hippocampus of the mouse brains through the Bcl-2/Bax pathway. J. Occup. Health.

[8] Chen F., Zhou C.C., Yang Y., Liu J.W., Yan C.H. (2019). GM1 Ameliorates Lead-Induced Cognitive Deficits and Brain Damage Through Activating the SIRT1/CREB/BDNF Pathway in the Developing Male Rat Hippocampus. Biol. Trace Elem. Res..

[9] Wang Y., Wang S., Cui W., He J., Wang Z., Yang X. (2013). Olive leaf extract inhibits lead poisoning-induced brain injury. Neural Regen. Res..

[10] Lan W., Lin J., Liu W., Wang F., Xie Y. (2021). Sulfiredoxin-1 protects spinal cord neurons against oxidative stress in the oxygen-glucose deprivation/reoxygenation model through the bax/cytochrome c/caspase 3 apoptosis pathway. Neurosci. Lett..

[11] Zhu X., Yao Y., Guo M., Li J., Yang P., Xu H., Lin D. (2021). Sevoflurane increases intracellular calcium to induce mitochondrial injury and neuroapoptosis. Toxicol. Lett..

[12] Kass G.E., Eriksson J.E., Weis M., Orrenius S., Chow S.C. (1996). Chromatin condensation during apoptosis requires ATP. Biochem. J..

[13] Imamura H., Sakamoto S., Yoshida T., Matsui Y., Penuela S., Laird D.W., Mizukami S., Kikuchi K., Kakizuka A. (2020). Single-cell dynamics of pannexin-1-facilitated programmed ATP loss during apoptosis. eLife.

[14] Luis-García E.R., Becerril C., Salgado-Aguayo A., Aparicio-Trejo O.E., Romero Y., Flores-Soto E., Mendoza-Milla C., Montaño M., Chagoya V., Pedraza-Chaverri J. (2021). Mitochondrial Dysfunction and Alterations in Mitochondrial Permeability Transition Pore (mPTP) Contribute to Apoptosis Resistance in Idiopathic Pulmonary Fibrosis Fibroblasts. Int. J. Mol. Sci..

[15] Nino S.A., Morales-Martinez A., Chi-Ahumada E., Carrizales L., Salgado-Delgado R., Perez-Severiano F., Diaz-Cintra S., Jimenez-Capdeville M.E., Zarazua S. (2019). Arsenic Exposure Contributes to the Bioenergetic Damage in an Alzheimer’s Disease Model. ACS Chem. Neurosci..

[16] Dua T.K., Dewanjee S., Gangopadhyay M., Khanra R., Zia-Ul-Haq M., De Feo V. (2015). Ameliorative effect of water spinach, Ipomea aquatica (Convolvulaceae), against experimentally induced arsenic toxicity. J. Transl. Med..

[17] Baldissarelli L.A., Capiotti K.M., Bogo M.R., Ghisleni G., Bonan C.D. (2012). Arsenic alters behavioral parameters and brain ectonucleotidases activities in zebrafish (*Danio rerio*). Comp. Biochem. Physiol. Part C Toxicol. Pharmacol..

[18] Dwivedi N., Mehta A., Yadav A., Binukumar B.K., Gill K.D., Flora S.J. (2011). MiADMSA reverses impaired mitochondrial energy metabolism and neuronal apoptotic cell death after arsenic exposure in rats. Toxicol. Appl. Pharmacol..

[19] Zheng X., Li S., Li J., Lv Y., Wang X., Wu P., Yang Q., Tang Y., Liu Y., Zhang Z. (2020). Hexavalent chromium induces renal apoptosis and autophagy via disordering the balance of mitochondrial dynamics in rats. Ecotoxicol. Environ. Saf..

[20] Yan X., Yu A., Zheng H., Wang S., He Y., Wang L. (2019). Calycosin-7-O-β-D-glucoside Attenuates OGD/R-Induced Damage by Preventing Oxidative Stress and Neuronal Apoptosis via the SIRT1/FOXO1/PGC-1α Pathway in HT22 Cells. Neural Plast..

[21] Yu Y., Zhao Y., Teng F., Li J., Guan Y., Xu J., Lv X., Guan F., Zhang M., Chen L. (2018). Berberine Improves Cognitive Deficiency and Muscular Dysfunction via Activation of the AMPK/SIRT1/PGC-1a Pathway in Skeletal Muscle from Naturally Aging Rats. J. Nutr. Health Aging.

[22] Zou Z., Hu X., Luo T., Ming Z., Luo Z. (2021). Naturally-occurring spinosyn A and its derivatives function as argininosuccinate synthase activator and tumor inhibitor. Nat. Commun..

[23] Guo X., Fu X., Liu X., Wang J., Li Z., Gao L., Li Y., Zhang W. (2019). Role of Pigment Epithelium-Derived Factor in Arsenic-Induced Vascular Endothelial Dysfunction in a Rat Model. Biol. Trace Elem. Res..

[24] Bai L., Tang Q., Zou Z., Meng P., Tu B., Xia Y., Cheng S., Zhang L., Yang K., Mu S. (2018). m6A Demethylase FTO Regulates Dopaminergic Neurotransmission Deficits Caused by Arsenite. Toxicol. Sci..

[25] Mehta K., Kaur B., Pandey K.K., Dhar P., Kaler S. (2021). Resveratrol protects against inorganic arsenic-induced oxidative damage and cytoarchitectural alterations in female mouse hippocampus. Acta Histochem..

[26] Li Y., Sun J., Wu R., Bai J., Hou Y., Zeng Y., Zhang Y., Wang X., Wang Z., Meng X. (2020). Mitochondrial MPTP: A Novel Target of Ethnomedicine for Stroke Treatment by Apoptosis Inhibition. Front. Pharmacol..

[27] Brenner C., Kroemer G. (2000). Apoptosis. Mitochondria--the death signal integrators. Science.

[28] Altznauer F., Conus S., Cavalli A., Folkers G., Simon H.U. (2004). Calpain-1 regulates Bax and subsequent Smac-dependent caspase-3 activation in neutrophil apoptosis. J. Biol. Chem..

[29] Binju M., Amaya-Padilla M.A., Wan G., Gunosewoyo H., Suryo Rahmanto Y., Yu Y. (2019). Therapeutic Inducers of Apoptosis in Ovarian Cancer. Cancers.

[30] Cregan S.P., MacLaurin J.G., Craig C.G., Robertson G.S., Nicholson D.W., Park D.S., Slack R.S. (1999). Bax-dependent caspase-3 activation is a key determinant in p53-induced apoptosis in neurons. J. Neurosci. Off. J. Soc. Neurosci..

[31] Won S.J., Kim D.Y., Gwag B.J. (2002). Cellular and molecular pathways of ischemic neuronal death. J. Biochem. Mol. Biol..

[32] Zhang W., Feng H., Gao Y., Sun L., Wang J., Li Y., Wang C., Zhao L., Hu X., Sun H. (2013). Role of pigment epithelium-derived factor (PEDF) in arsenic-induced cell apoptosis of liver and brain in a rat model. Biol. Trace Elem. Res..

[33] Radajewska A., Szyller J., Niewiadomska J., Noszczyk-Nowak A., Bil-Lula I. (2023). Punica granatum L. Polyphenolic Extract as an Antioxidant to Prevent Kidney Injury in Metabolic Syndrome Rats. Oxid Med Cell Longev..

[34] Du J., Hang P., Pan Y., Feng B., Zheng Y., Chen T., Zhao L., Du Z. (2019). Inhibition of miR-23a attenuates doxorubicin-induced mitochondria-dependent cardiomyocyte apoptosis by targeting the PGC-1α/Drp1 pathway. Toxicol. Appl. Pharmacol..

[35] Jia N., Sun Q., Su Q., Dang S., Chen G. (2016). Taurine promotes cognitive function in prenatally stressed juvenile rats via activating the Akt-CREB-PGC1α pathway. Redox Biol..

[36] Prakash C., Kumar V. (2016). Arsenic-induced mitochondrial oxidative damage is mediated by decreased PGC-1α expression and its downstream targets in rat brain. Chem.-Biol. Interact..

[37] Halling J.F., Pilegaard H. (2020). PGC-1α-mediated regulation of mitochondrial function and physiological implications. Appl. Physiol. Nutr. Metab..

[38] Navazani P., Vaseghi S., Hashemi M., Shafaati M.R., Nasehi M. (2021). Effects of Treadmill Exercise on the Expression Level of BAX, BAD, BCL-2, BCL-XL, TFAM, and PGC-1α in the Hippocampus of Thimerosal-Treated Rats. Neurotox. Res..

[39] Tritos N.A., Mastaitis J.W., Kokkotou E.G., Puigserver P., Spiegelman B.M., Maratos-Flier E. (2003). Characterization of the peroxisome proliferator activated receptor coactivator 1 alpha (PGC 1alpha) expression in the murine brain. Brain Res..

[40] Chen S.D., Yang D.I., Lin T.K., Shaw F.Z., Liou C.W., Chuang Y.C. (2011). Roles of oxidative stress, apoptosis, PGC-1α and mitochondrial biogenesis in cerebral ischemia. Int. J. Mol. Sci..

[41] Szalardy L., Zadori D., Plangar I., Vecsei L., Weydt P., Ludolph A.C., Klivenyi P., Kovacs G.G. (2013). Neuropathology of partial PGC-1α deficiency recapitulates features of mitochondrial encephalopathies but not of neurodegenerative diseases. Neurodegener. Dis..

[42] St-Pierre J., Drori S., Uldry M., Silvaggi J.M., Rhee J., Jager S., Handschin C., Zheng K., Lin J., Yang W. (2006). Suppression of reactive oxygen species and neurodegeneration by the PGC-1 transcriptional coactivators. Cell.

[43] Zhang Q., Lei Y.H., Zhou J.P., Hou Y.Y., Meng H. (2019). Role of PGC-1α in Mitochondrial Quality Control in Neurodegenerative Diseases. Neurochem. Res..

[44] Chen Z., Tao S., Li X., Yao Q. (2018). Resistin destroys mitochondrial biogenesis by inhibiting the PGC-1α/NRF1/TFAM signaling pathway. Biochem. Biophys. Res. Commun..

[45] Mostafa Tork O., Ahmed Rashed L., Bakr Sadek N., Abdel-Tawab M.S. (2019). Targeting Altered Mitochondrial Biogenesis in the Brain of Diabetic Rats: Potential Effect of Pioglitazone and Exendin-4. Rep. Biochem. Mol. Biol..

[46] Nasehi M., Torabinejad S., Hashemi M., Vaseghi S., Zarrindast M.R. (2020). Effect of cholestasis and NeuroAid treatment on the expression of Bax, Bcl-2, Pgc-1α and Tfam genes involved in apoptosis and mitochondrial biogenesis in the striatum of male rats. Metab. Brain Dis..

[47] Zhang X., Ren X., Zhang Q., Li Z., Ma S., Bao J., Li Z., Bai X., Zheng L., Zhang Z. (2016). PGC-1α/ERRα-Sirt3 Pathway Regulates DAergic Neuronal Death by Directly Deacetylating SOD2 and ATP Synthase β. Antioxid. Redox.

[48] Yang L., Jiang Y., Xiaoqian Y.E., You Y., Lin L., Lian J., Juan L.I., Yang S., Xue X. (2022). The neuroprotection of electro-acupuncture via the PGC-1α/TFAM pathway in transient focal cerebral ischemia rats. Biocell.

